# Quantum model for supercontinuum generation process

**DOI:** 10.1038/s41598-022-13808-8

**Published:** 2022-06-11

**Authors:** A. Safaei Bezgabadi, M. A. Bolorizadeh

**Affiliations:** 1 Department of Nanotechnology, Graduate University of Advanced Technology, P.O. Box: 76315-117, Kerman, Iran; 2 Department of Photonics, Graduate University of Advanced Technology, Kerman, Iran

**Keywords:** Solitons, Supercontinuum generation, Quantum physics

## Abstract

A quantum theory is established for the propagation of electromagnetic waves in highly nonlinear dispersive optical fibers. By applying the method recently presented dispersion terms and retarded response of the medium are included for the propagation of light in a fiber in this work. A coupled stochastic generalized nonlinear Schrödinger equation (GNLSE) is obtained via the coherent positive-P representation to describe the supercontinuum generation process. This coupled quantum-stochastic equation is applied to obtain the linearized fluctuation equation for studying quantum noise and the fluctuation in the vicinity of the formed solitons in the supercontinuum generation process in the region of anomalous dispersion. Also, these equations can be used to study the soliton self-frequency shift quantum mechanically. Finally, we simulate the obtained coupled stochastic generalized nonlinear Schrödinger in the mean case and compare our simulation results with experimental results.

## Introduction

The study of nonlinear optics (NLO) gained strong momentum^1^ as new high power light sources were invented^2^. There has been both classical^3,4^ and quantum^5–9^ treatment of nonlinear optics as well as some experimental reports^10,11^ in literature. NLO was studied during the first two decades after the invention of laser, before being ignored in the 1980s^12^. During the following decades, the main goal of scientists was to prevent the negative effects of nonlinear optical deficiencies, especially in fiber communications^13–15^. However, by the turn of century, nonlinear optical effects began to be engineered towards the advancement of optical systems^15^. Many research groups have implemented quantum theories for the propagation of the electromagnetic waves through dielectrics^9,16–18^. In the earlier studies, the dielectrics were considered to be non-dispersive and free of nonlinear response. Hillery^19^ initiated a theory for non-dispersive media taking into account an instantaneous nonlinear response. Implementing Hillery’s method for nonlinear media, Drummond^8^ worked out a quantum theory for the field propagation through a nonlinear dispersive dielectric. He assumed an instantaneous nonlinear response for the medium, considering the dispersion up to the second order term. Carter^20^ presented a quantum theory for the pulse propagation along optical fiber considering several Hamiltonians extending the previous quantum theories for pulse propagation through optical fibers. Many researchers investigated the generation of quantum states by optical waveguides and using them for diverse applications, including quantum non-demolition experiments^21^, generation of Schrödinger’s cat states^22^, parametric down conversion^23^, photonic quantum technologies^24^, and novel generation of optical sources like optical frequency combs based on micro-resonators^25^.

The process by which a high intensity light pulse is launched into a dispersive dielectric, the extreme frequency broadening is observed due to dispersive and nonlinear effects, known as supercontinuum generation^26^. Both experimental and theoretical studies performed by several groups indicate the formation of the supercontinuum light in a dielectric; e.g. optical fiber. A comprehensive study of this process was published by Dudley et al.^27^ based on classical models. Supercontinuum light sources have influenced the advancement of science and technology due to their widespread applications^26–35^.

Previously, we have presented a preliminary report^36^ on the quantum treatment of radiation near the zero dispersion wavelengths of the fiber. The quantum theories used to date for the propagation of electromagnetic wave omit the higher order dispersion terms and the dispersive nature of the nonlinear response^9,19,36–40^. We have developed a theory for canonical quantization of the radiation in the presence of the third order dispersion term^41^. Making use of this quantization scheme and considering that the non-dispersive and instantaneous assumptions of nonlinear response are not suitable for ultra-short pulses, we quantized the supercontinuum generation process. Our previous report, exhibits that the supercontinuum generation is not supported by a model where retarded response of the medium is not taken into account^37^. In this work, we will focus on the quantum treatment of supercontinuum generation by electromagnetic radiation in the presence of the higher order dispersion terms, retarded response of the medium and dispersion of the third-order susceptibility.

The main cause of instability in a dielectric used to generate supercontinuum light is noise which should be reduced in industry applications^30,42,43^. Depending on the parameters of the input pulse, the generated supercontinuum noise could cause up to 50% fluctuation in the temporal intensity profile of the output pulse^30,43^. The fundamental part of this noise is quantum noise, which is inherent in the nonlinear process leading to the generated continuum light. Corwin and coworkers measured the noise and provided a model to describe it^43^. Their model, a semi-classical one, adds a noise term into the generalized nonlinear Schrödinger equation (GNLSE). The term added to GNLSE, by Corwin et al.^43^, is a quantum noise, which has been phenomenologically a sound term to be added to the classical GNLSE. Real-time measurements of optical noise showed long-tailed statistics in the spectral intensity of a generated supercontinuum light^44–46^. Gonzalo et al.^47^ were able to reduce noise fluctuation in all-normal dispersion supercontinuum sources. However, the instability of the supercontinuum light due to the noise is still an open question.

There are a vast number of studies on the supercontinuum light sources based on classical electromagnetism. However, there has been growing research works in exploring their applications in quantum optics. The quantum treatment of supercontinuum is essential for the development of modern (non-classical) light sources where they are attracting great interest in quantum information, quantum communications, and on-chip quantum devices such as integrated single- or entangled- photon sources^48–54^.

We intend to devise a quantum mechanical model to treat the supercontinuum generation in a microstructure fiber. This model enables us to study the soliton self-frequency shift. Nonetheless, the noise in this kind of solitons could be dealt with by employing this quantum mechanical formalism. The quantum treatment of supercontinuum phenomenon is essential not only for the progress in understanding NLO where quantum conditions hold, but also for the development of quantum communication, quantum information, and spectroscopy. In addition, there are fluctuations around the formed solitons in this process, which could be due to quantum noises^55^. The higher order solitons split into lower order and fundamental ones due to the soliton interactions, the higher order dispersion effect, the Raman effect and the self-steepening phenomenon^56^. A powerful quantum theory is needed to explain all these effects collectively. In order to examine the quantum aspects of supercontinuum generation, a detailed formulation containing all effective phenomena governing the supercontinuum generation will be presented using the positive-P representation^57,58^.

## Theory

We choose the usual model for a centrosymmetric highly nonlinear fiber (photonic crystal fiber (PCF)), where the first nonlinear term to be included is the third order nonlinear susceptibility (*χ*^(3)^), which is not instantaneous in all practical applications (for details, see Ref.^28^). In order to quantize the pulse propagation in optical fibers, it is necessary to find a canonical Lagrangian leading to the correct Hamiltonian and to the Maxwell’s equations as the Hamilton’s equation of motion. It should be emphasized that in the present work, we include the higher order dispersion terms and the retarded medium response employing the approach recently developed^41^. The present method follows the method developed by Drummond^8,9^. One could obtain the Lagrangian and define appropriate creation and annihilation operators applying the method introduced in Ref.^41^, which satisfy the harmonic oscillator commutation relations. The Hamiltonian is:

1 $$\hat{H} = \sum\limits_{k} {\hbar \omega (k){\hat{a}}_{k}^{\dag } {\hat{a}}_{k} } - \frac{{\varepsilon_{0} \chi^{(3)} }}{{4\varepsilon^{4} }}\int {\left( {\hat{D}^{2} \left( {t,{\mathbf{x}}} \right)\int_{ - \infty }^{t} {R(t^{\prime})\,\hat{D}^{2} \left( {t - t^{\prime},{\mathbf{x}}} \right)\,dt^{\prime}} } \right)d^{3} {\mathbf{x}}}$$

where ${\hat{a}}_{k}$, $\hat{D}$, $\varepsilon_{0}$, $\varepsilon$, and R(t) are mode operators, electric displacement operator, vacuum permittivity, medium permittivity, and response function of the medium (which is absent in Drummond’s works^8,9,59,60^ and our earlier works^36,40,41^), respectively. The first term in Eq. (1) which was determined earlier in terms of creation and annihilation operators derived when higher order dispersion term included in the Lagrangian^41^ differs from those obtained by Drummond^9^. We also assume the interaction picture and discretize the spatial and wave number dependence; i.e. $k \equiv k_{n} = k_{0} + n\Delta k$. The expansion of ω(k) in Taylor series near k_0_ leads to:

2 $$\omega \left( k \right) = \omega \left( {k_{0} } \right) + \left( {k - k_{0} } \right)\omega^{\prime} + \frac{1}{2}\left( {k - k_{0} } \right)^{2} \omega^{\prime\prime} + \frac{1}{6}\left( {k - k_{0} } \right)^{3} \omega^{\prime\prime\prime} + \cdots$$

where $\omega^{\prime} = {{d\omega } \mathord{\left/ {\vphantom {{d\omega } {dk}}} \right. \kern-\nulldelimiterspace} {dk}}|_{{k = k_{0} }}$, $\omega^{\prime\prime} = {{d^{2} \omega } \mathord{\left/ {\vphantom {{d^{2} \omega } {dk^{2} }}} \right. \kern-\nulldelimiterspace} {dk^{2} }}|_{{k = k_{0} }}$ and $\omega^{\prime\prime\prime} = {{d^{3} \omega } \mathord{\left/ {\vphantom {{d^{3} \omega } {dk^{3} }}} \right. \kern-\nulldelimiterspace} {dk^{3} }}|_{{k = k_{0} }}$ (and so on) correspond to the group velocity, group velocity dispersion and the third order dispersion coefficient, respectively. When the wavelength of the input pulse is close to the fiber’s zero dispersion wavelengths, the third order dispersion coefficient is dominant. Note that, the third order dispersion coefficient is zero for dispersion flattened fibers operated in the flat region of dispersion curve. Therefore, this work is irrelevant to the dispersion flattened fibers.

Due to the discrete nature of the longitudinal mode spacing ($\Delta k = {{2\pi } \mathord{\left/ {\vphantom {{2\pi } L}} \right. \kern-\nulldelimiterspace} L}$**)** in a fiber of length L, for convenience one can define the local operators^59,60^:

3 $$\hat{\alpha }_{\ell } \triangleq (2N + 1)^{{ - {1 \mathord{\left/ {\vphantom {1 2}} \right. \kern-\nulldelimiterspace} 2}}} \sum\nolimits_{n = - N}^{N} {{\hat{a}}_{n} \,\exp \left( {{{2\pi ni\ell } \mathord{\left/ {\vphantom {{2\pi ni\ell } {(2N + 1)}}} \right. \kern-\nulldelimiterspace} {(2N + 1)}}} \right)}$$

where spatial z-dependence, (${\text{z}} \equiv \ell \Delta {\text{z}}$), is discrete and the steps of $\Delta z = {L \mathord{\left/ {\vphantom {L {(2N + 1)}}} \right. \kern-\nulldelimiterspace} {(2N + 1)}}$ are arranged along the fiber from $\ell = - N$ to + N. These operators satisfy the harmonic oscillator commutation relations^59,60^. The quantum mechanical treatment of the supercontinuum generation process is done by making use of the positive-P representation. We did not follow the Glauber-Sudarshan P representation^61,62^, which is not suitable here as it leads to a Fokker–Planck equation with non-positive definite diffusion coefficients. Rather, we follow the method developed by Drummond^57,58^ and start with the definition^20,59^:

4 $$\left| {\left. {\theta (t)} \right\rangle } \right.\left\langle {\theta (t)} \right| = \int {P(t;{{\varvec{\upalpha}}},{{\varvec{\upalpha}}}^{ + } )\frac{{\left| {{\varvec{\upalpha}}} \right\rangle \left\langle {({{\varvec{\upalpha}}}^{ + } )^{*} } \right|}}{{\left\langle {{({{\varvec{\upalpha}}}^{ + } )^{*} }} \mathrel{\left | {\vphantom {{({{\varvec{\upalpha}}}^{ + } )^{*} } {{\varvec{\upalpha}}}}} \right. \kern-\nulldelimiterspace} {{{\varvec{\upalpha}}}} \right\rangle }}} \,d^{2} {{\varvec{\upalpha}}}d^{2} {{\varvec{\upalpha}}}^{ + }$$

where $\left| {{\varvec{\upalpha}}} \right\rangle$ and $\left| {\left. {\theta (t)} \right\rangle } \right.$ are the coherent state of total field and the total wave function for the interaction Hamiltonian, respectively. In addition, $P(t;{{\varvec{\upalpha}}},{{\varvec{\upalpha}}}^{ + } )$ develops in time according to a Fokker–Planck equation with positive semidefinite diffusion coefficients. The Fokker–Planck equation amounts to equations of motion for the eigenvalues of the local operators $\alpha_{\ell }^{{}}$ and $\alpha_{\ell }^{ + }$ (components of appropriate stochastic variables ${{\varvec{\upalpha}}}(t)$ and ${{\varvec{\upalpha}}}^{ + } (t)$, respectively) are:

5a $$\begin{aligned} \frac{{\partial \alpha_{\ell } }}{\partial t} & = i\left( {\chi_{\alpha } \alpha_{\ell } \left[ {\int_{ - \infty }^{t} {R(t^{\prime})\,\alpha_{\ell }^{ + } (t - t^{\prime})\alpha_{\ell } (t - t^{\prime})\,dt^{\prime}} } \right] + \chi_{\alpha } \alpha_{\ell }^{ + } \alpha_{\ell } \left[ {\int_{ - \infty }^{t} {R(t^{\prime})\,\alpha_{\ell } (t - t^{\prime})\,dt^{\prime}} } \right] - \sum\limits_{\ell ^{\prime}} {\omega_{\ell \ell ^{\prime}} \alpha_{\ell ^{\prime}} } } \right) \hfill \\ & \quad + \left( {2i\chi_{\alpha } } \right)^{{{1 \mathord{\left/ {\vphantom {1 2}} \right. \kern-\nulldelimiterspace} 2}}} \alpha_{\ell } \,\xi_{\ell } (t) \hfill \\ \end{aligned}$$

and

5b $$\begin{aligned} \frac{{\partial \alpha_{\ell }^{ + } }}{\partial t} & = - i\left( {\chi_{\alpha } \alpha_{\ell }^{ + } \left[ {\int_{ - \infty }^{t} {R(t^{\prime})\,\alpha_{\ell }^{ + } (t - t^{\prime})\alpha_{\ell } (t - t^{\prime})\,dt^{\prime}} } \right] + \chi_{\alpha } \alpha_{\ell }^{ + } \alpha_{\ell } \left[ {\int_{ - \infty }^{t} {R(t^{\prime})\,\alpha_{\ell }^{ + } (t - t^{\prime})\,dt^{\prime}} } \right] - \sum\limits_{\ell ^{\prime}} {\omega_{\ell \ell ^{\prime}} \alpha_{\ell }^{ + } } } \right)\hfill \\ & \quad + \left( { - 2i\chi_{\alpha } } \right)^{{{1 \mathord{\left/ {\vphantom {1 2}} \right. \kern-\nulldelimiterspace} 2}}} \alpha_{\ell }^{ + } \,\xi_{\ell }^{ + } (t) \hfill \\ \end{aligned}$$

where

5c $$\omega_{\ell \ell ^{\prime}} = \sum\limits_{n} {\left( {2N + 1} \right)^{ - 1} \left[ {\left( {n\Delta k} \right)\omega^{\prime} + \frac{1}{2}\left( {n\Delta k} \right)^{2} \omega^{\prime\prime} + \frac{1}{6}\left( {n\Delta k} \right)^{3} \omega^{\prime\prime\prime} + \cdots } \right]} \times \exp \left[ {\frac{2\pi ni}{{2N + 1}}\left( {\ell - \ell ^{\prime}} \right)} \right]\,$$

and

5d $$\chi_{\alpha } = \frac{{3\varepsilon_{0} \hbar \left( {\omega^{\prime}k_{0} } \right)^{2} }}{{8\varepsilon^{2} \Delta V}}\chi^{(3)}$$

Here, $\xi_{\ell } (t)$ and $\xi_{\ell }^{ + } (t)$ are real Gaussian stochastic functions with the correlation relations $\left\langle {\xi_{\ell } (t_{1} )\xi_{\ell ^{\prime}}^{ + } (t_{2} )} \right\rangle = 0$ and $\left\langle {\xi_{\ell } (t_{1} )\xi_{\ell ^{\prime}} (t_{2} )} \right\rangle = \left\langle {\xi_{\ell }^{ + } (t_{1} )\xi_{\ell ^{\prime}}^{ + } (t_{2} )} \right\rangle = \delta_{\ell \ell ^{\prime}} \delta (t_{1} - t_{2} )$. Note firstly, that the rotating wave approximation is used to calculate the integral term of the Hamiltonian (1) and secondly, $\alpha_{\ell }$ and $\alpha_{\ell }^{ + }$ are not exactly complex conjugate to each other as we made use of the positive-P representation. The stochastic field, $\Phi$, is defined as^59,60^:

6 $$\Phi (z) \triangleq \mathop {\lim }\limits_{\Delta z \to 0} \left( {\alpha_{\ell } } \right)\left( {\frac{{\omega^{\prime}}}{\Delta z}} \right)^{{{1 \mathord{\left/ {\vphantom {1 2}} \right. \kern-\nulldelimiterspace} 2}}}$$

in the limit $\Delta z \to 0$, at the location ${\text{z}} \equiv \, \ell \Delta {\text{z}}$. As the continuum representation is now used, the discrete stochastic terms are replaced by Gaussian stochastic fields $\xi (T,z)$ and $\xi^{ + } (T,z)$, with correlation relations

7a $$\left\langle {\xi (T_{1} ,z_{1} )\xi_{\ell ^{\prime}} (T_{2} ,z_{2} )} \right\rangle = \left\langle {\xi_{{}}^{ + } (T_{1} ,z_{1} )\xi_{{}}^{ + } (T_{2} ,z_{2} )} \right\rangle = \delta (z_{1} - z_{2} )\delta (T_{1} - T_{2} )$$

and

7b $$\left\langle {\xi (T_{1} ,z_{1} )\xi_{{}}^{ + } (T_{2} ,z_{2} )} \right\rangle = 0.$$

In addition to the quantum noise, one may include additional fluctuations due to variations in the refractive index, which would result in a set of correlation relation different from those of Eq. (7). Making use of the transformation ($T = t - {z \mathord{\left/ {\vphantom {z {\omega^{\prime}}}} \right. \kern-\nulldelimiterspace} {\omega^{\prime}}}$), the problem is solved in a new frame moving at a velocity equal to the group velocity. Let’s define a wavenumber dependent nonlinear parameter, $\chi_{\Phi }$, which is defined similar to $\chi_{\alpha }$. For all practical cases, it can be expanded as $\chi_{\Phi } = \chi_{\Phi } \left( {k_{0} } \right) + \left( {k - k_{0} } \right)\chi^{\prime}_{\Phi } + \cdots$ where $\chi^{\prime}_{\Phi } = \left. {{{d\chi_{\Phi } } \mathord{\left/ {\vphantom {{d\chi_{\Phi } } {dk}}} \right. \kern-\nulldelimiterspace} {dk}}} \right|_{{k = k_{0} }}$.

Finally, the full stochastic equation governing the supercontinuum generation process for the field Ф(T,z) is obtained as:

8a $$\frac{\partial }{\partial z}\Phi (T,z) = \left( { - \frac{{i\omega^{\prime\prime}}}{{2\omega^{{\prime}{3}} }}\frac{{\partial^{2} }}{{\partial T^{2} }} + \frac{{\omega^{\prime\prime\prime}}}{{6\omega^{{\prime}{4}} }}\frac{{\partial^{3} }}{{\partial T^{3} }} + \cdots } \right)\Phi (T,z) + i\left( {\chi_{\Phi } + i\chi^{\prime}_{\Phi } \frac{\partial }{\partial T}} \right)\Psi (T,z) + \left( {i\chi_{\Phi } } \right)^{{{1 \mathord{\left/ {\vphantom {1 2}} \right. \kern-\nulldelimiterspace} 2}}} \xi (T,z)\Phi (T,z)$$

where

8b $$\Psi (T,z) = \Phi (T,z)\,\left[ {\int_{ - \infty }^{t} {R(T^{\prime})\,\Phi^{ + } (T - T^{\prime},z)\,\Phi (T - T^{\prime},z)\,dT^{\prime}} } \right] + \Phi^{ + } (T,z)\Phi (T,z)\,\left[ {\int_{ - \infty }^{t} {R(T^{\prime})\,\Phi (T - T^{\prime},z)\,dT^{\prime}} } \right]$$

There is also a coupled equation to the Eq. (8a) by transforming $\Phi \to \Phi^{ + }$, $i \to - i$ and $\xi \to \xi^{ + }$, which in the mean case it is the complex conjugate of Eq. (8a). Equation (8a) can be regarded as the fundamental result of the present work. Equation (8) together with the coupled equation for $\Phi^{ + } (T,z)$ form a set of coupled generalized nonlinear Schrödinger equations for the quantum treatment of the supercontinuum generation in fibers (e.g. PCF), which we call it coupled quantum-stochastic GNLSE. Here, in addition to the stochastic terms which introduce fluctuations originated from quantum noise and coupling form of the two equations, the main difference between the coupled quantum-stochastic equations (Eq. 8) and its classical form (i.e. GNLSE) is the term proportional to $\int_{ - \infty }^{t} {R(T^{\prime})\Phi (T - T^{\prime},z)dT^{\prime}}$ in Eq. (8) which has no counterpart in classical forms. This additional term is brought about by commutation relations, which holds for creation and annihilation operators. Indeed, this term appears when one obtains the Fokker–Planck equation (from master equation) by using Hamiltonian (1). Also, note that the quantity $\chi_{\Phi }$ introduced here for the quantum case is half its value for the classical form.

Assuming higher order dispersion terms are ignored in the instantaneous medium response limit, R(T) is replaced by delta function where the quantity defined by Eq. (8b) takes the form $\Psi (T,z) = 2\Phi^{ + } (T,z)\,\Phi^{2} (T,z)$. Therefore, the coupled quantum-stochastic equation (Eq. 8a) leads to the well-known coupled stochastic nonlinear Schrödinger equation resulted from the Fokker–Planck equation which is a rigorous basis for results of pioneering works^9,19,60,63^ and our previous research^36^. Also, if one does not use the nature of the commutation relation in the calculations, the resulted equation of motion (master equation) from Hamiltonian (1) leads to the classical generalized nonlinear Schrödinger equation^26–28^.

There are self-phase modulation and cross-phase modulation, which are the side effect of Kerr effect and four wave mixing due to the $\chi_{\Phi }$. Also, the stimulated Raman scattering and self-steepening are discussed assuming the retarded response function, R(T), and the dispersive nature of $\chi_{\Phi }$, ($\chi^{\prime}_{\Phi } {\partial \mathord{\left/ {\vphantom {\partial {\partial T}}} \right. \kern-\nulldelimiterspace} {\partial T}}$).

The coupled quantum-stochastic equations will have soliton solutions, called quantum solitons^59,60^. There are many works in the literature to study quantum solitons^9,10,20,22,55,64–66^, but these solitons are not studied in supercontinuum generation process and in these works higher order dispersion coefficients are not included. If these solitons are not fundamental ones, they split into the lower order and fundamental ones after propagating inside the fiber, which is known as soliton fission. In some applications of the supercontinuum generation (e.g. quantitative experiments), it is necessary to squeeze the quantum noise in the vicinity of these solitons.

In order to investigate the squeezing of these fluctuations, we use the definitions and results of pioneering researchers on the topic of squeezing of quantum solitons^59,60^. For the spectrum of quadrature fluctuations in the field, they expressed that relatively small quantum fluctuations can be approximated by a linearized fluctuation equation^59,60^. Hence, the linearized fluctuation equation to study quantum noise will be:

9 $$\begin{gathered} \frac{\partial }{\partial z}\delta \Phi (T,z) = \left( { - \frac{{i\omega^{\prime\prime}}}{{2\omega^{{\prime}{3}} }}\frac{{\partial^{2} }}{{\partial T^{2} }} + \frac{{\omega^{\prime\prime\prime}}}{{6\omega^{{\prime}{4}} }}\frac{{\partial^{3} }}{{\partial T^{3} }} + \cdots } \right)\delta \Phi (T,z) + i\chi_{\Phi } \left( {\int_{ - \infty }^{t} {R(T^{\prime})\phi_{0}^{2} (T - T^{\prime})dT^{\prime}} } \right)\delta \Phi (T,z) + \hfill \\ + i\chi_{\Phi } \phi_{0} (T)\left( {\int_{ - \infty }^{t} {R(T^{\prime})\phi_{0} (T - T^{\prime})dT^{\prime}} } \right)\delta \Phi (T,z) + i\chi_{\Phi } \phi_{0}^{2} (T)\int_{ - \infty }^{t} {R(T^{\prime})\delta \Phi (T - T^{\prime},z)dT^{\prime}} \hfill \\ + i\chi_{\Phi } \phi_{0} (T)\int_{ - \infty }^{t} {R(T^{\prime})\phi_{0} (T - T^{\prime})\delta \Phi (T - T^{\prime},z)dT^{\prime}} + i\chi_{\Phi } \phi_{0} (T)\delta \Phi^{ + } (T,z)\int_{ - \infty }^{t} {R(T^{\prime})\phi_{0} (T - T^{\prime})dT^{\prime}} \hfill \\ + i\chi_{\Phi } \phi_{0} (T)\int_{ - \infty }^{t} {R(T^{\prime})\phi_{0} (T - T^{\prime})\delta \Phi^{ + } (T - T^{\prime},z)dT^{\prime}} + \left( {i\chi_{\Phi } } \right)^{1/2} \phi_{0} (T)\xi (T,z) \hfill \\ \end{gathered}$$

where $\phi_{0} (T,z) = \left\langle {\Phi (T,z)} \right\rangle$ and $\Phi (T,z) = \phi_{0} (T,z) + \delta \Phi (T,z)$. We recall that the governing equations are the Eq. (9) together with the equation that will be obtained by using the transformation $\Phi \to \Phi^{ + }$, $i \to - i$ and $\xi \to \xi^{ + }$, which is called the coupled quantum-stochastic noise equations.

Here, ϕ_0_ can be considered as a mean case solution to the coupled generalized nonlinear Schrödinger equations for the quantum treatment of the supercontinuum generation (Eq. (8)). The function ϕ_0_ at z = 0 corresponds to a classical coherent input state, that in resemblance to the classical case, can be treated as $\phi_{0} (T,z = 0) = \sqrt {P_{0} } {\text{sech}} (T/T_{0} )$, where P_0_ and T_0_ are, respectively, peak power and width of the input pulse which is launched into the photonic crystal fiber. In the instantaneous medium response limit where R(T) is replaced by delta function (together with ignoring higher order dispersion terms), Eq. (9) is reduced to the linearized fluctuation equation obtained in Ref.^36^ where it was numerically solved in Ref.^67^ and has properly shown the quantum fluctuations for the propagating solitons in an optical fiber in the presence of the second and third order dispersion coefficients.

In summary, a quantum description of the supercontinuum generation in pulse propagation through a highly nonlinear fiber is established. This theory leads to a coupled quantum-stochastic generalized nonlinear Schrödinger equation. In addition to the coupling term, the second term in Eq. (8b) was also obtained to describe the supercontinuum generation that is not present in the classical generalized nonlinear Schrödinger equation. The reason behind this difference is the commutation relation that holds for the stochastic fields of the master equation, leading to the Fokker–Planck equation.

Making use of a stochastic field, the quantum noise source was included in the governing equation. Subsequently, the coupled linearized fluctuation equation is obtained by implementing the proper definition for squeezed quantum solitons^59,60^. One argues that the resulted squeezing for the normal dispersion regime is different from the resultant squeezing for the anomalous dispersion regime. In order to arrive at this prediction, it is necessary to solve the coupled linearized fluctuation equation numerically. The equations, obtained here, could be used to study non-optical systems involving third order retarded response, when the Hamiltonian (1) holds.

## Simulation results and discussion

Applying the 4th order Runge–Kutta algorithm and employing the quantum treatment, Eq. (8), the supercontinuum generation in photonic crystal fiber under the mean case can be studied where $\Phi$ and $\Phi^{ + }$ are complex conjugates to each other. Note that the expectation value of the last term in Eq. (8a) is zero under the mean case^9,60^.

Indeed, the implemented numerical algorithm to solve Eq. (8) is the same as the process for solving common GNLSE (classical treatment). Several numerical methods have been developed for solving the GNLSE equation^26,28,68,69^. For simulating both quantum-GNLSE and common GNLSE, the Fourier transform and inverse Fourier transform (i.e. transformed the equation to the frequency domain then transformed back to the time domain) are applied in each integration step to treat the dispersion terms (the terms contain time derivatives). Also, the integral terms (convolution integrals) present in these equations can be evaluated accordingly by transforming each functions in the integrands into the frequency domain, multiplying, then transforming back^26,28,68,69^. Here, under the mean case condition, the main difference between two treatments is the additional term in Eq. (8) which is brought about by commutation relations between the creation and annihilation operators that this term is not exist in common GNLSE.

**Figure 1:**
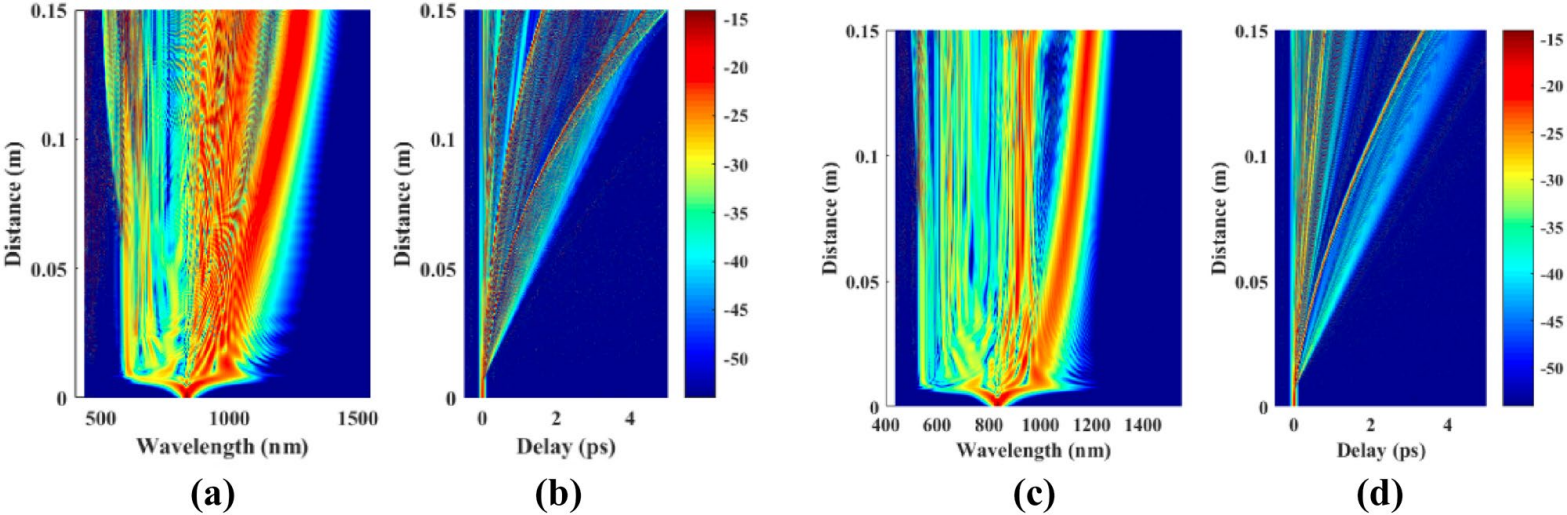
Simulation results for the quantum mechanical (**a** and **b**) and classical treatment (c and d) of the supercontinuum generation, SCG, along the first 15 cm of a PCF fiber. Photons generated at wavelengths from 400 nm (**a**) to 1450 nm in a quantum treatment and (**c**) to 1350 nm in a classical treatment of SCG. Time development of the input pulse is shown in (**b**) and (**d**) with respect to the delayed pulse in a quantum and classical models, respectively.

The present quantum mechanical model of the supercontinuum generation is compared with the experimental results of Dudley and his group^27,43^ assuming a PCF illuminated by a defined incoming light. The simulated results, the quantum and the classical treatments, for the retarded nonlinear response condition are shown in Fig. 1. The input pulse of 10 kW peak power and 28.4 fs width at 835 nm^27^ was chosen in this work. Due to the additional term in Eq. (8), which is absent in the classical GNLSE, there are differences between the classical GNLSE and the quantum mechanical treatment of the supercontinuum generation. Note that the higher order dispersion terms and retarded response of the medium is not included in the work reported by reference^37^ and therefore the results do not show the soliton self-frequency shift and soliton fission present here.

**Figure 2:**
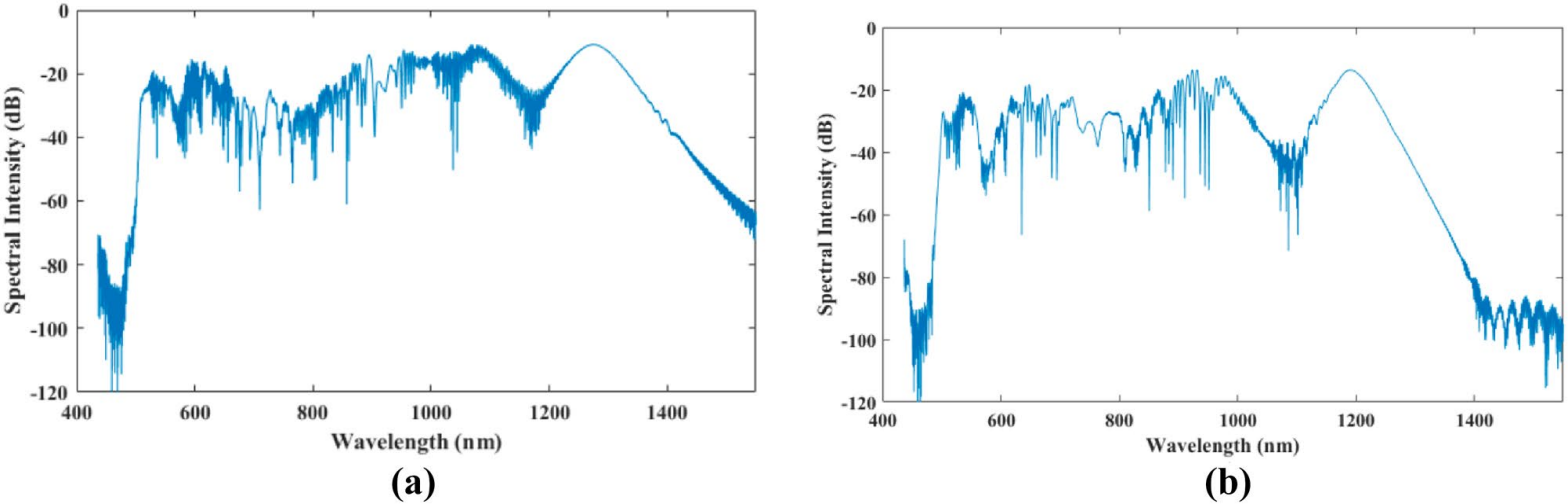
The generated wavelengths at the output of a 15 cm PCF in (**a**) quantum mechanical and (**b**) classical calculations for the input pulse of 10 kW power and 28.4 fs pulse width at 835 nm.

As compared with the results of the classical treatment of the supercontinuum generation in a specific PCF shown by Figs. 1c,d and 2b, the present quantum results presented in Figs. 1a,b and 2a, show that not only does the spectral broadening increases, but also that the generated frequency combs are closer and richer. In Fig. 1b, the quantum mechanical model predicts longer delay for the formed soliton as compared with the classical result of Fig. 1d. However, the two models predict the soliton fission to occur at about the same travel distance of the pulse along the fiber.

As described earlier, the additional term in Eq. (8) is originated from the non-commutative nature of the creation and the annihilation operators defined in obtaining the Fokker–Planck equation. This term changes the evolution of the optical pulse inside the fiber, which causes the difference in the simulation results between the two treatments. In reality, this term alters how the nonlinear part would affect the broadening of the pulse and generation of new frequencies. As an example, the soliton evolves due to the interplay between the dispersion terms, the Raman scattering term, and the self-steepening term. Thus the evolution of solitons is predicted differently in the quantum treatment as compared with the classical model.

**Figure 3:**
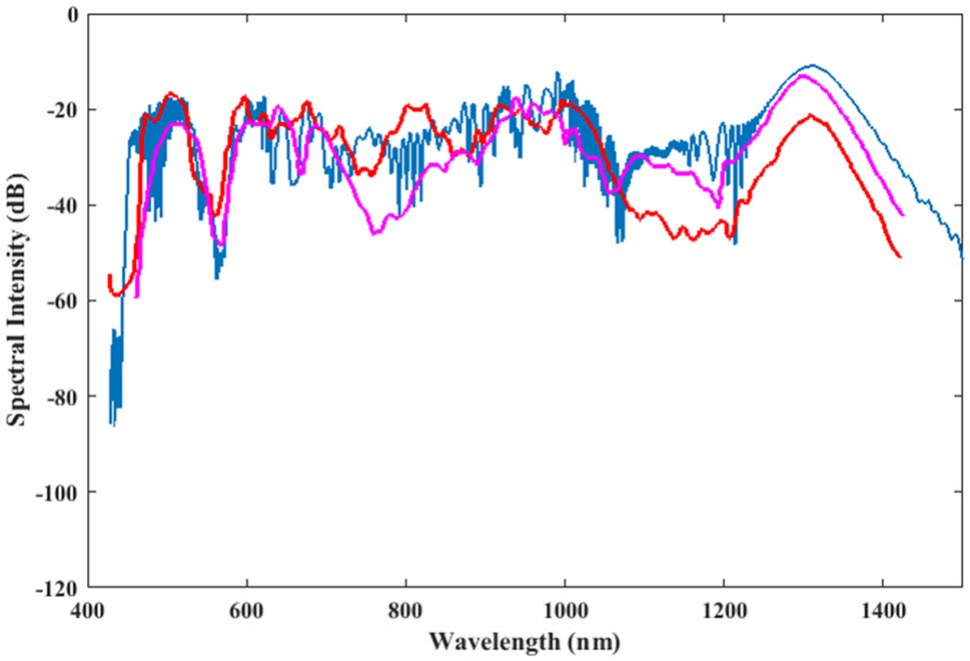
A comparison between the present simulation of supercontinuum generation (the blue line), the experimental results (the red line) and the simulation results (the purple line) of Corwin et al.^43^.

From a methodological standpoint, the present simulation results are compared with the available experimental work^43^ for a 0.9 nJ pulse at 18 fs pulse width (22 fs full width at half maximum) which measured the supercontinuum spectrum between 400 to 1400 nm as shown in Fig. 3. Figure 3 indicates that the peak of soliton in experimental results agrees with the quantum mechanical model ones. In an experimental work, the resolution of the spectrometer should be less than 1 nm, in order to be able to show the frequency (wavelength) combs and deeper details of the continuum generation. Even though the present simulation results have wavelength steps of 1.12 nm, the wavelength combs are not clear in some regions. We therefore suggest a more detailed and precise experimental work needed in this field to be able to understand the deeper physics behind the nonlinear effects in dielectrics and, especially, fibers. This is essential in order to understand and to reduce the noise and most important, the quantum noise. The agreement between the present simulation results and those of the experimental data in the region 450–1050 nm favors the approach presented in Ref.^43^. For example, this is specifically evident as the red line (experimental data) crosses over the quantum simulation results in the region of 850–1050 nm. Our quantum simulation results are much closer to the experimental ones in the region 750–850 nm and around 500 nm as compared with the theoretical approach of^43^. As indicated from Fig. 3, the intensity of soliton peak in the simulation results is higher than the experimental one. This is partly due to the fact that a lossless PCF is assumed, while in reality each medium exhibits some sort of loss. In our model we have considered all physical phenomena that had been included in the work of Ref^43^ except the loss because our focus is on the main phenomena which have most contributions on the supercontinuum process. The theoretical approach used in Ref.^43^ included the loss, so the frequency width of the Raman soliton is less than that in or simulation results. The loss can also affect the intensity of the spectrum. If the loss is added to the present quantum model, the intensity of the Raman soliton will be closer to its experimental intensity.

**Figure 4:**
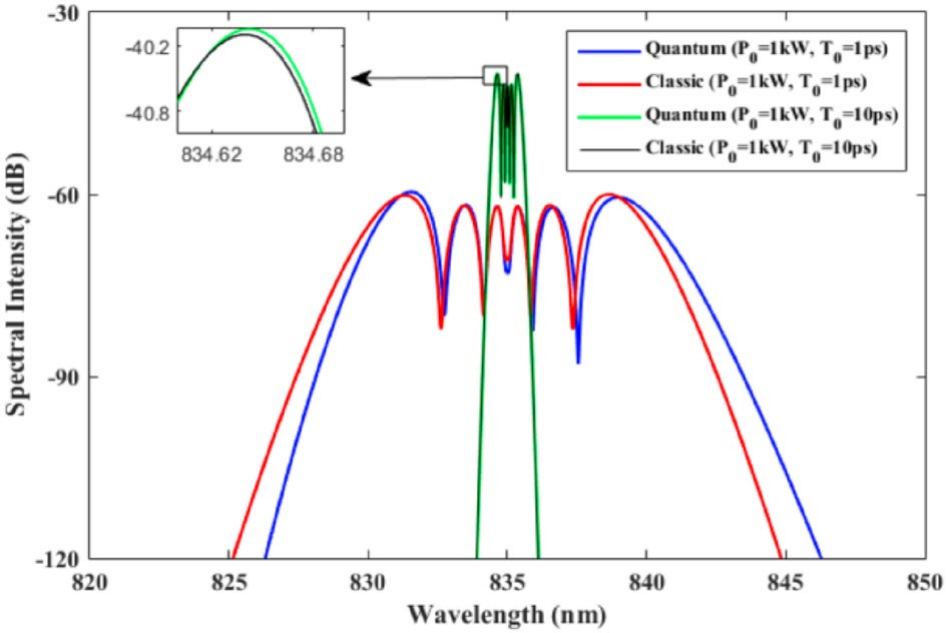
The propagation of light pulses at 1 kW, low power, and pulse width of 1 ps and 10 ps are compared for both classical and quantum mechanical models. Inset shows details of the spectrum in a region of the spectrum.

In the following, the quantum mechanical treatment results are compared with the classical ones for different peak powers and pulse widths. Figure 4 indicates that when the peak power is low, such as 1 kW, and the pulse width is large, 10 ps, the two models lead, approximately, to similar results in the mean case. When the quantum nature of incoming light pulse is not measureable, the classical and quantum treatments are not distinguishable. Actually, to verify the presented quantum model, the appropriate apparatus and detection schemes must be used for quantum measurement of SC generation spectrum as discussed in Ref.^70^ for quantum measurement of an optical frequency comb. Note that the quantum mechanical description of wave and particles are the same and leads to the wave packet descriptions of both. When the width of a light pulse is small, it represents a small particle which makes the quantum treatment essential. It is interesting that the two results, classical and quantum model, deviate as the width of input pulse changes from 10 to 1 ps. The classical results are closer to the quantum mechanical model at the pulse width of 10 ps as compared with the 1 ps pulses. This study shows how important is the quantum nature of electromagnetic pulses at short pulse width. In practice, the nonlinear terms do not have significant contributions on the generation of pulses of new wavelengths at such peak powers and pulse widths. However, the quantum mechanical treatment of the pulse propagation along the optical fiber is still indispensable, because this approach is applicable to study quantum solitons and to reduce the fluctuations in the vicinity of these solitons. Soliton, a solitary wave, could be considered as a collection of particles traveling together in a medium from a quantum mechanical point of view.

It is noted that, the input can be considered as a macroscopic quantum state containing a cluster of approximately 10^9^ photons. By our quantum model, the photon number of each state, phase fluctuation and related Heisenberg uncertainties can be discussed theoretically, however; further measurements based on quantum measurement theory are needed to validate them. Here, in order to show our model is consistent with the classical measurement of SCG, we simulated Eq. (8) in mean case.

## Conclusion

In conclusion, none of the previous quantum theoretical treatments of light pulses passing through a nonlinear media describes the supercontinuum generation process. Here, the quantum theory for the supercontinuum generation process is presented, and it has included the terms with the significant contributions involved in the supercontinuum generation in a nonlinear media, specifically the PCFs. Besides the obtained coupled stochastic equation for the quantum treatment of the supercontinuum generation process, the source of significant difference is the additional term which has no classical resemblance and roots in the non-commutative nature of the creation and the annihilation operators defined in obtaining the Fokker–Planck equation. The result of the quantum treatment, as compared with the experimental results, indicates conformity theory and experiment^43^. The generated supercontinuum spectrums for the quantum mechanical and the classical treatments are studied for different peak powers and widths of the input pulse. It is concluded that the pulses of fs width behave as particles and described best by quantum models.

There are different types of noise involved in supercontinuum light, or generally in the propagation of electromagnetic waves in a nonlinear media, which are related to absorption, gain, Raman effects, and the quantum mechanical noise that could be described by stochastic equations. The noise involved in Raman effects has quantum origin and it is present in a vacuum state^43^ due to spontaneous Raman effects. A semi-classical model of noise, due to the propagation of light pulses in fibers devised by Corwin and coworkers^43^, is based on adding this kind of noise to the GNLSE. However, the second order nonlinear term was not included which is the source of the Raman noise. Nonetheless, this noise exists and should be included in the quantum theory of the field propagation in dielectric media^30,43–47^. In the present work, in order to reduce the fluctuations near solitons, one has to make use of Eq. (9) instead of Eq. (8), a process that is referred to as linearization^59^. However, it should be noted that the linearization is handled for squeezed photons and, therefore, Eq. (9) is not valid for long − distance travel of light pulses even for an instantaneous nonlinear response. Therefore, what was discussed, here, includes the quantum mechanical noise involved in the stochastic equations. Also, it is important to add the quantum noise involved in Raman effects to Eq. (8) to reduce the quantum noises in the vicinity of the solitons, which were formed during the supercontinuum generation process. New experimental work based on real-time measurement, similar to those of Närhi et al.^46^ and Wetzel et al.^45^, is needed to study the supercontinuum generation and all noises involved. Detailed understanding of the physics of ultrashort light pulses needs more experimental works and their comparison with the present quantum theory of pulse propagation, specifically in fiber applications. Nonetheless, the medium response function and/or the parameters involved in it (such as Raman parameters and Raman delayed factor) could be different when a light pulse should be modeled quantum mechanically.

## Data Availability

The datasets used and/or analysed during the current study available from the corresponding author on reasonable request.
